# Natural variation in *Arabidopsis thaliana* rosette area unveils new genes involved in plant development

**DOI:** 10.1038/s41598-020-74723-4

**Published:** 2020-10-19

**Authors:** Rubén González, Anamarija Butković, Mark Paul Selda Rivarez, Santiago F. Elena

**Affiliations:** 1 Instituto de Biología Integrativa de Sistemas (I2SysBio), CSIC-Universitat de València, Parc Cientific UV, Catedrático Agustín Escardino 9, Paterna, 46980 Valencia, Spain; 2 The Santa Fe Institute, 1399 Hyde Park Road, Santa Fe, NM 87501 USA; 3 Present Address: Department of Biotechnology and Systems Biology, National Institute of Biology, Večna pot 111, 1000 Ljubljana, Slovenia

**Keywords:** Genetics, Plant sciences

## Abstract

Growth is a complex trait influenced by multiple genes that act at different moments during the development of an organism. This makes it difficult to spot its underlying genetic mechanisms. Since plant growth is intimately related to the effective leaf surface area (*ELSA*), identifying genes controlling this trait will shed light on our understanding of plant growth. To find new genes with a significant contribution to plant growth, here we used the natural variation in *Arabidopsis thaliana* to perform a genome-wide association study of *ELSA*. To do this, the projected rosette area of 710 worldwide distributed natural accessions was measured and analyzed using the genome-wide efficient mixed model association algorithm. From this analysis, ten genes were identified having SNPs with a significant association with *ELSA*. To validate the implication of these genes into *A. thaliana* growth, six of them were further studied by phenotyping knock-out mutant plants. It was observed that *rem1.2*, *orc1a*, *ppd1*, and *mcm4* mutants showed different degrees of reduction in rosette size, thus confirming the role of these genes in plant growth. Our study identified genes already known to be involved in plant growth but also assigned this role, for the first time, to other genes.

## Introduction

Growth is a complex highly polygenic trait, controlled by many small-effect loci contributing to quantitative trait variation^1^. Growth processes in plants, such as increase in leaf size, also have a temporal component as they involve genes at specific developmental stages playing specific or general roles^2^. Moreover, plant growth is not only affected by internal factors but also by external ones: since plants are sessile organisms, their traits are strongly influenced by environmental factors such as light^3,4^,water status^5^ or availability of mineral resources^6^. As an example, the wide diversity in leaf patterns illustrates how the dynamics in timing and environmental conditions affect the expression of genes involved in the process^7,8^.

Since the past decade there has been an increase in the number of quantitative plant genes for traits as flowering time, growth or plant defense. However there has been limitations dissecting these traits as they integrate multiple mechanisms controlled by many loci^9^. Thus, the complex genetic basis of growth necessarily implies that hundreds of variants that control the trait would not be easily identified in genome-wide association studies (GWAS)^10^. This explains why the set of growth-related genes described to have a significant effect in the leaf phenotype and their functional roles remains poorly studied. For example, the flower and fruit development genetics and epigenetics are more studied in comparation with leaf development^11^. This is, among other causes, due to the difficulty in differentiating and describing individual processes in leaf growth because multiple gene activation and cell differentiation events happen sequentially or almost at the same time in the leaf primordia or meristem^12^. Nevertheless, technological and computational advances in GWAS and phenomics have made significant strides. This has helped to reveal previously unknown genes or genomic regions tightly associated with measurable leaf growth traits such as leaf area and biomass^13,14^. At present, there is certain knowledge on gene regulatory modules that are involved in the cell proliferation leading to size expansion namely, *ENHANCER OF DA1* (*EOD1*), *GROWTH REGULATING FACTOR 1* (*GRF1*), *GRF1-INTERACTING FACTOR 1* (*GIF1*), *SWITCH/SUCROSE NONFERMENTING 3C* (*SWI3C*), *GIBBERELLIC ACID INSENSITIVE* (*GA1*), *CYTOCHROME P450 78A POLYPEPTIDE 5* (*KLU*), and *PEAPOD 1* and* 2* (*PPD1*, *PPD2*)^15^. Experimental data and network analysis showed that these modules are directly linked to the cell cycle machinery and regulation, but there are still gaps in the process that need to be studied^16^.

Most functional genetic studies in plants now follow the top-down reverse genetics approach to uncover multiple genes involved in complex phenotypes^17^. In this work, we use this approach on *Arabidopsis thaliana* (L.) Heynh plants. *A. thaliana* is a prime model organism in plant and crop genetics and genomics research^18^ with a natural genetic and phenotypic variation that has allowed the analysis of multiple traits related to growth^19^. In *A. thaliana* the leaves form a rosette at the base of the plant, so the projected area of the rosette will be equal to the photosynthetically effective leaf surface area of the plant during its vegetative growth. Even after flowering, the plant will only have a few leaves on the stem, concentrating most of its leaf area in the rosette^20^. Therefore, even though plant growth can be described through multiple traits (e.g., rosette size, plant height and weight, number of lateral branches, or number of leaves) the projected leaf area is a relevant one when measuring growth as this trait is key in plants for the amount of light intercepted, which is tightly correlated to plant productivity^21^. In addition, plant mass positively correlates with projected and total leaf area during plant development, despite these traits having a linear relationship only during the vegetative growth stage. This is because after flowering the relocation of carbon to inflorescence growth also plays an important role^22^. As measuring the leaf area was complicated in the past, other traits such as rosette diameter and weight were measured instead. Nowadays, the improvement and simplification of the technology needed for leaf area measurement makes it possible to estimate the surface of the leaf through non-invasive digital images^23^. These images can be processed in real-time, obtaining area values for the horizontal projection of the leaves, which is the area capable of light interception or the effective leaf surface area (*ELSA*)^24^.

Here, we used digital images to measure the rosette *ELSA* of a set of 710 *A. thaliana* natural accessions. Owed to *A. thaliana* variation for leaf rosette, we were able to collect a set of data diverse enough to perform a GWAS. The GWAS has allowed the identification of loci significant for *ELSA* and are probably involved in rosette growth. Moreover, we used knock-out (KO) mutants of candidate genes identified in our GWAS that are readily available in seed banks. This allowed immediate validation of their contribution to the *ELSA* phenotype. Finally, our experiments and subsequent functional genome exploration in public databases showed that a set of previously known and unknown genes have a significant contribution into the leaf rosette *ELSA* phenotype. This set of unknown genes thus warrants further functional experimental studies.

## Results

### Rosette *ELSA* data variation correlates with latitude

The projected rosette area of natural accessions grown at 24 °C in 16 h long days were measured 45 days after sowing (das). A total of 710 accessions were studied, from which 189 had flowered by the time of the measurement, so their stem was removed prior to measuring the rosette’s *ELSA*.

**Figure 1:**
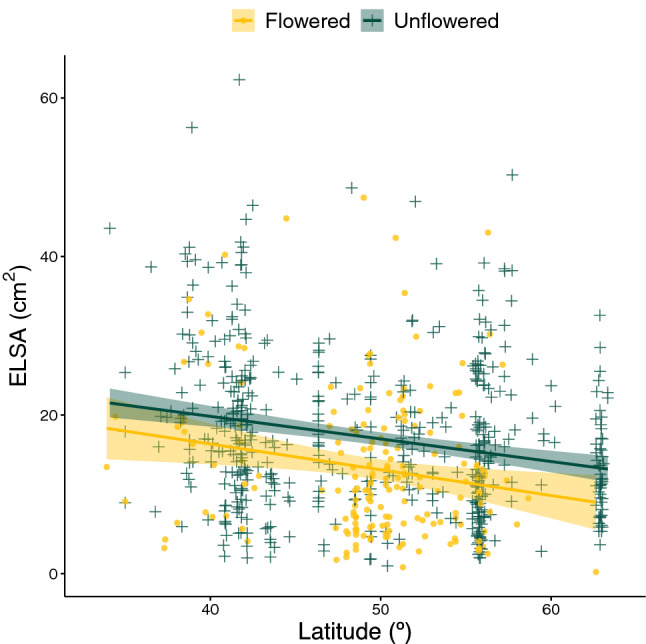
Rosette’s *ELSA* correlation with latitude. Data of flowered plants are indicated in yellow and of unflowered ones in green. Regression lines are represented by solid lines, whereas shadowed areas represent the 95% CI of the regression lines.

We found a significant correlation of the rosette *ELSA* with the latitudinal origin of the natural accession (Fig. 1). The higher the latitude the more reduced rosette *ELSA*, this reduction being independent of whether the plant had flowered or not by the time the measures were taken. The Pearson correlation of the *ELSA* with the latitude for flowered natural accessions was *r* =  − 0.1918 (*t*_187_ =  − 2.6721, *P* = 0.0082) and *r* =  − 0.2296 for unflowered ones (*t*_519_ =  − 5.3732, *P* = 0.0001). By contrast, we did not find any correlation of the rosette *ELSA* with the longitudinal origin of the *A. thaliana* natural accessions, neither for the flowered (*t*_187_ =  − 1.0885, *P* = 0.2778) or the unflowered ones (*t*_519_ = 0.3417, *P* = 0.7327). Furthermore, an analysis of covariance (ANCOVA) using flowering status (flowered or unflowered) as a binary factor and latitude as a continuous covariable shows that there are no significant differences in *ELSA* associated to the flowering status (*F*_1,706_ = 0.0741, *P* = 0.7856, ${\eta }_{P}^{2}$ = 0.0001). Likewise, there was no significant interaction between the flowering status and the latitude (*F*_1,706_ = 0.0841, *P* = 0.7719, ${\eta }_{P}^{2}$ = 0.0001), thus resulting in parallel regression lines (Fig. 1). Finally, the latitude itself has a small yet highly significant effect on *ELSA* (*F*_1,706_ = 19.3517, *P* = 0.0001, ${\eta }_{P}^{2}$ = 0.0267), supporting the conclusions based in correlation analyses. Notice that, hereafter, the magnitude of effects was evaluated using the ${\eta }_{P}^{2}$ statistic (proportion of total variability in the observed variable attributable to each factor in the model under consideration); conventionally, values of ${\eta }_{P}^{2}$ < 0.05 are considered as small, 0.05 ≤ ${\eta }_{P}^{2}$  < 0.15 as moderate and ≥ ${\eta }_{P}^{2}$0.15 as large effects.

In conclusion, rosette’s *ELSA* values do not depend on whether plants have flowered or not but vary along a latitudinal cline: the more northern the origin of a natural accession, the smaller *ELSA* it has.

### GWAS identifies genetic loci associated with rosette area

The rosette’s *ELSA* data were standardized according to their flowering status (flowered or unflowered) and an association study was performed with the resulting standardized values using genome-wide efficient mixed model association algorithm (GEMMA)^25^. Thirty single nucleotide polymorphism (SNP) were detected with a significance higher than the established threshold − log*P* > 5, some of these SNPs being positioned within the genes that are showed in Table 1.

**Table 1:** Significant SNPS (− log*P* > 5) detected in the GWAS.

Gene		Description	− log*P*	Chromosome	SNP position
*AT4G14713	*PPD1*	Protein TIFY 4A	5.9394	4	8,427,519
*AT2G16440	*MCM4*	Minichromosome maintenance 4	5.8632	2	7,128,128
*AT2G42710*	–	Ribosomal protein L1p/L10e family	5.8337	2	17,783,903
*AT4G14700	*ORC1A*	Origin of replication complex 1	5.7126	4	8,423,290
*AT4G06210*	–	Natural antisense transcript overlaps with *AT4G14700*	5.7126	4	8,423,290
*AT4G34000	*ABF3*	Abscisic acid responsive elements-binding factor 3	5.3617	4	16,298,093
*AT1G75200	–	Flavodoxin family protein	5.1492	1	28,222,144
*AT5G59610*	*DJC73*	Chaperone DnaJ-domain superfamily protein	5.1451	5	2,4012,081
*AT5G59600*	–	Pentatricopeptide repeat-containing protein	5.1451	5	24,012,081
*AT3G61260	*REM1.2*	Remorin family protein	5.0854	3	22,676,514

Genes marked with an asterisk were selected for further study in order to validate the results.

**Figure 2:**
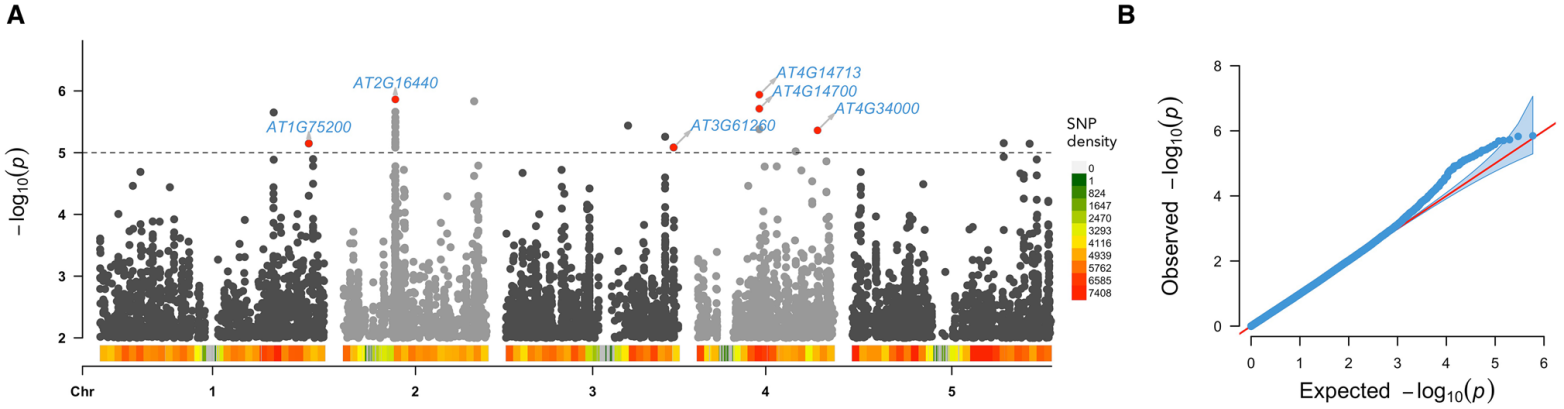
(**A**) Manhattan plot of GWAS results for the *ELSA* phenotype. Red dots represent the most significant SNPs of the genes (marked by arrows) that would be further analyzed using KO mutants. (**B**) QQ-plots show significant SNPs in the tail peak; population structure inflation is not observed. The 95% confidence interval is shaded in blue. Plots were done using the R package “rMVP” v1.0.0 (https://github.com/xiaolei-lab/rMVP).

The SNPs and their associated *P* values are plotted in Fig. 2A, together with the genes selected for a follow-up study of homozygotic KO plants. The quantile–quantile plot (QQ-plot) shows that there is no population structure inflation (Fig. 2B). The heritability of the *ELSA* trait was estimated from GEMMA analysis and from Bayesian sparse linear mixed model (BSLMM) because the latter can explain variation in highly polygenic traits as well as traits with a few genes of large effect. Chip heritability or SNP based heritability is the proportion of phenotypic variation that can be explained by genotyped genetic markers (PVE). PVE using BSLMM and LMM was similar (0.2364) indicating that 23.64% of the phenotypic variance is explained by the 591,427 genotyped SNPs. By contrast, the proportion of genetic variance explained by sparse effect loci is referred to as PGE. The number of variants with large effects size among the 591,427 SNPs was 42 and these major effect loci (PGE) explained 47% of PVE. This relatively low PGE and the relatively large number of variants with significant effect means that their overall effect sizes are small, indicating that the trait is controlled by many small effect variants.

### Validation of significance in selected genes

**Figure 3:**
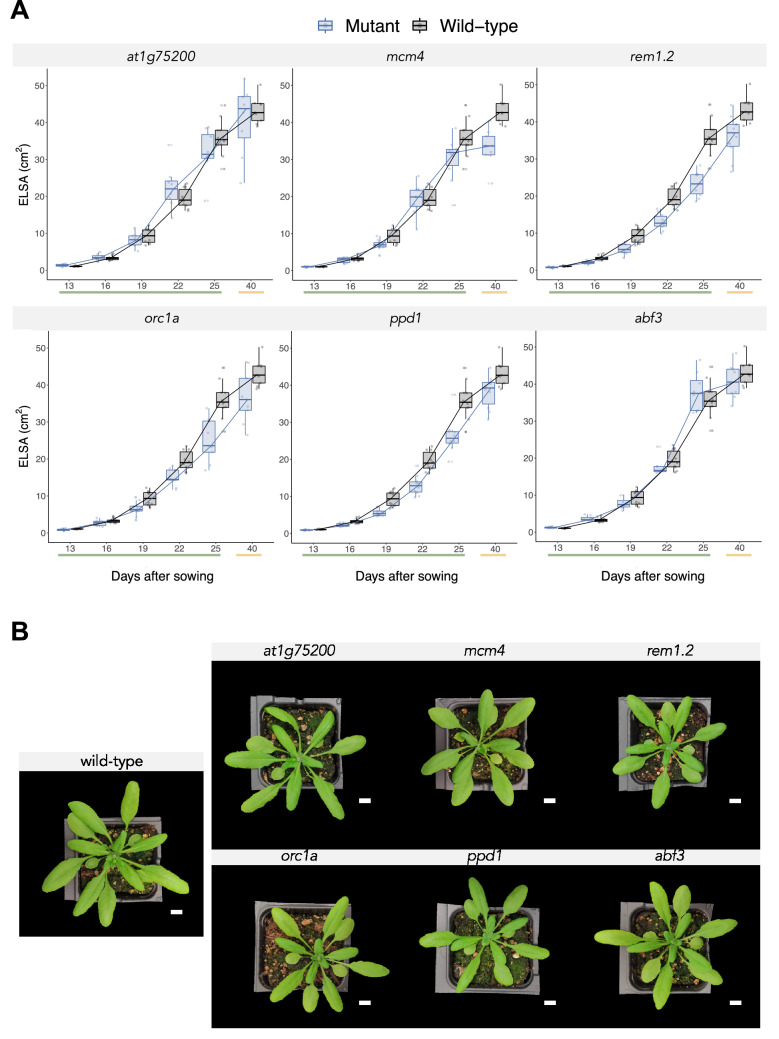
(**A**) Temporal dynamics of rosette’s *ELSA* during plant growth for the indicated gene KO mutants (blue) compared with the wild-type (black). The measures include growth at the vegetative state (green underlined) and days after the flowering initiation of the plants (yellow underlined). (**B**) Pictures of the wild-type and mutant plants at 27 das. White bar indicates 1 cm.

To confirm the effect of the above candidate genes in rosette growth, we compared the rosette’s *ELSA* of mutants (Col-0 plants with a KO gene) with wild-type plants. To do so, we measured the rosette’s *ELSA* of the plants during their vegetative growth (13, 16, 19, 22, and 25 das) and after the flowering initiation (40 das). The comparation of the measurements between wild-type and the KO plants is shown in Fig. 3A.

Analyzing the complete time series performing a repeated-measures ANOVA (with plant genotype as inter-individual factor and das as intra-individual factor) we found a significant difference in the *ELSA* of the plants (Wilks’ *Λ* = 0.1171, *F*_30,218_ = 5.1566, *P* = 0.0001, ${\eta }_{P}^{2}$ = 0.3489). Post hoc comparisons with Bonferroni correction indicated that *rem1.2* (mean difference ± 1 SD =  − 5.4201 ± 1.0046 cm^2^, *P* = 0.0001), *orc1a* (− 4.4353 ± 1.0321 cm^2^, *P* = 0.0014) and *ppd1* (− 4.5348 ± 1.0046 cm^2^, *P* = 0.0007) had a significantly reduced *ELSA* in comparation with wild-type plants. At late growth stages, some mutants seemed to show a reduction of their rosette’s *ELSA*, therefore, a second repeated-measures ANOVA was performed using the measurements at 25 and 40 das. This analysis provided similar results (*Λ* = 0.7909, *F*_6,58_ = 2.5520, *P* = 0.0280, ${\eta }_{P}^{2}$ = 0.2091) but the pairwise Bonferroni post hoc comparations found four mutants showing a significative difference between the mutant and the wild-type plants: *mcm4* (− 7.4605 ± 2.079 cm^2^, *P* = 0.0144) and the three identified in the complete time series *rem1.2* (− 10.5280 ± 2.0794 cm^2^, *P* = 0.0001), *orc1a* (− 9.2877 ± 2.1364 cm^2^, *P* = 0.0011) and *ppd1* (− 7.9155 ± 2.0794 cm^2^, *P* = 0.0071). This reduction in the *ELSA* was not accompanied by alterations in their leaf shape or morphology as all KO mutants show a wild-type-like contour (Fig. 3B).

Rosette’s *ELSA* was measured at 40 das, a few days after plants end vegetative growth. To quantitatively determine the end of the vegetative growth we used the day plants bolted. A one-way ANOVA comparing the wild-type (mean ± 1 SD = 26.6000 ± 0.5164 das) and the mutants showed an overall difference in bolting times of very large magnitude (*F*_6,58_ = 7.2497, *P* = 0.0001, ${\eta }_{P}^{2}$ = 0.4286). The Bonferroni post hoc test showed that *at1g75200* (27.0000 ± 1.1952 das), *mcm4* (27.2000 ± 1.6193 das) and *abf3* (27.5000 ± 0.9258 das) had no significant difference in bolting time. The mutants *rem1.2* (28.5000 ± 1.1785 das, *P* = 0.0123), *orc1a* (28.5556 ± 1.333 das, *P* = 0.0121) and *ppd1* (29.4000 ± 1.0750 das, *P* = 0.0001) had a significant delay in their bolting time.

**Figure 4:**
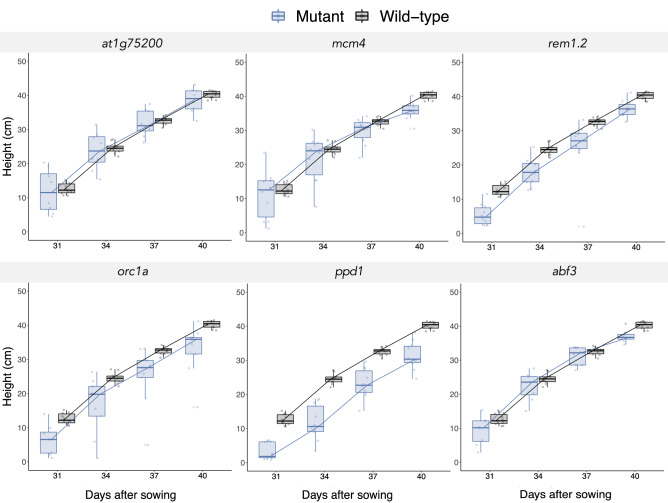
Temporal dynamics of the height for the indicated gene KO mutants (blue) compared with the wild-type (black).

After bolting, the plant height was evaluated by measuring their stem at days 31, 34, 37, and 40 das (Fig. 4). By performing a repeated-measures ANOVA we found a significant large effect in the height of the plants, (*Λ* = 0.4326, *F*_18,153.22_ = 2.9349, *P* = 0.0002, ${\eta }_{P}^{2}$ = 0.2437).

The pairwise Bonferroni post hoc tests showed the height dynamics of the *rem1.2* (mean difference ± 1 SD =  − 6.2850 ± 1.7197 cm, *P* = 0.0119) and *ppd1* (− 9.4836 ± 1.7668 cm, *P* = 0.0001) had significant reduction in comparation with the wild-type plants. The rest of the KO mutants did not show significant differences in height compared with wild-type plants.

The observation of the selected six KO phenotypes indicates that, under our experimental conditions, four of them have a reduction in their rosette’s *ELSA*. On the one hand a reduction in the rosette’s *ELSA* was observed in the late growth of *mcm4* plants but their bolting time and height was undistinguishable from wild-type plants. On the other hand, *orc1a* plants had a reduced *ELSA* during all its growth and bolted later than wild-type plants but their height was the same. Finally, *rem1.2* and *ppd1* showed a reduced *ELSA* during all its growth, a late bolting and a smaller height compared to wild-type.

## Discussion

Natural variation has allowed finding genes underlying complex plant, helping to understand developmental processes (e.g., seed germination, vegetative growth, flowering, or morphology) and plant physiology^26^. *A. thaliana* shows an impressive natural variation, partly due adaptation to its wide climatic amplitude, from North Scandinavia to Tanzania and Kenya mountains^19^. For this study we have analyzed the rosette area variation of accessions with a latitudinal distribution from 33.92° to 63.32°. Selected natural accessions were all grown under the same temperature and long day conditions so, even outside their presumable optimum, the accessions *ELSA* correlated with the latitude from which they came from. This has also been proved for flowering, which was found to be influenced by the latitude even after removing the effect of climatological factors^27^. Li et al.^28^ studied plant size of a smaller set of *A. thaliana* (40 natural accessions) and also found that plants from higher latitudes tended to have smaller leaf areas. To further characterize the *ELSA* we have studied its heritability, which is 23.64%. This is in line with previous studies, which have shown that traits related with flowering have heritability higher than 80% but for biomass accumulation related traits it is in the range of 20–60%^10^.

We have been able of measuring *ELSA* in a large set of *A. thaliana* natural accessions thanks to the advances in image processing technology. The Easy Leaf Area program^23^ has allowed us to measure the trait in an intuitive, accurate, low cost and non-invasive way as it only needs digital images taken with a smartphone. The availability of naturally occurring inbred lines of *A. thaliana* makes GWAS a good approach for unveiling the genetics of plant traits^29^. The information and resources available for this model organism also facilitate this sort of experimental approach. In the first GWAS done with *A. thaliana*, different traits were measured in 96–192 natural accessions^30^. Thanks to the 1001 *Arabidopsis* genome consortium^31^ now it is possible to perform studies with hundreds of natural accessions and GWAS has become an essential tool for disentangling the genetic basis of *A. thaliana* complex traits. For example, Kooke et al.^14^ analyzed 349 natural accessions for developmental traits (leaf area, relative growth rate, flowering time, leaf length, and petiole length) to spot 30 candidate genes with SNPs showing − log*P* > 4. One of these genes, involved in the ratio of the leaf length and petiole length, was validated *in planta* for leaf morphology but no further study about leaf area was done. Bac-Molenaar et al.^2^ studied growth dynamics of 324 accessions and using a more stringent cut-off, − log*P* > 5, identified 26 SNPs highly associated with one or more of the traits they studied (fresh weight, projected leaf area and parameters derived from a growth model). No further experimental validation was done. None of the genes identified in the studies of Kooke et al.^14^ and Bac-Molenaar et al.^2^ match the ones identified in the present study. This is not surprising since no single gene has a major contribution to growth and the measures differ from ours: Kooke et al.^14^ evaluated rosette leaf area of plant growth at days 15, 19, 63, and 68 days after germination while Bac-Molenaar et al.^2^ measured the rosette leaf area at multiple time points until day 28 after being transferred to the growth chamber. Growth conditions of Kooke et al.^14^ and Bac-Molenaar et al.^2^ (16 h light day, 125 µmol m^−2^ s^−1^, 70% RH, 20/18 °C day/night cycle) also differ from ours in relative humidity and temperature (45% RH, 24/20 °C day/night cycle). As in these previous two studies, we have used an arbitrary cut-off since leaf area is a polygenic trait and the stringency of Bonferroni thresholds for some GWAS impedes the discovery of loci truly implicated in the trait^32^. To reduce possible false discoveries, we established a stringent threshold of − log*P* > 5 and further validated six of the identified loci by studying the phenotypes of the corresponding KO mutants.

We have detected multiple significant loci in the *ELSA* GWAS analysis, with 30 of them positioned within 10 genes. From the identified genes *AT2G42710*, *AT4G06210*, *AT5G59610*, and *AT5G59600* KO mutants were not further evaluated. *AT2G42710* encodes for a ribosomal protein of the L1p/L10e family mainly expressed in the mitochondria but its function is not defined. Still other mitochondria ribosomal proteins have a role in plant development as mutants lacking some of these proteins have been shown to have affected their embryo, vegetative or reproductive development^33^. The identification of *AT2G42710* in this *ELSA* GWAS suggest that this mitochondria-ribosomal protein is involved in vegetative development. *AT4G06210* is a natural antisense transcript overlapping with *ORIGIN OF REPLICATION COMPLEX 1* (*ORC1A*) gene involved in plant growth that will be discussed below. *AT5G59600* encodes for a protein that contains tetratricopeptide repeat (TPR) motifs and it may participate in diverse biological processes such as cellular biosynthesis but also plant secondary metabolism, such as response to abiotic stimuli^34^. The identification of *AT5G59600* in our *ELSA* GWAS suggest the gene is involved in cellular biosynthesis as this function will be implicated in growth. *AT5G59610* locus corresponds to the *CHAPERONE DNA J PROTEIN C73* (*DJC73*) gene that encodes for a J-domain-containing chaperone mainly expressed in chloroplasts. DJC73 function is unknown but as a chloroplast J protein its functions may be related to biosynthesis^35^ and therefore may have some implication in plant growth.

The other six genes were experimentally evaluated by measuring the growth of their KO mutants. *AT1G75200* and *ABSCISIC ACID RESPONSIVE ELEMENT-BINDING FACTOR 3* (*ABF3*) did not show any significant effect in the plant growth under our experimental conditions. *AT1G75200* encoded protein is a flavodoxin, and it is involved in a response to multiple stresses and iron deficiency^36^. Therefore, the disruption of this gene did not affect plant development under optimal growth conditions. It is a possibility that *AT1G75200* regulates growth only during conditions of abiotic stress. For *abf3* plants we failed to observe any significant change in *ELSA*, an observation at odds with Wang et al.^37^ report that *ABF3* overexpression reduced the leaf size in alfalfa. *ABF3* may have a contribution to *A. thaliana* growth that is not sufficient by itself to be detected in our KO mutants experimental set up.

We have identified two genes that have been previously described as involved in growth. These genes are *ORIGIN RECOGNITION COMPLEX 1A* (*ORC1A*) and *PPD1*, whose KOs showed a significant reduction in *ELSA*, a smaller height and a late bolting in our experimental set up. ORC1A along with the product of gene *ORIGIN RECOGNITION COMPLEX 1B* (*ORC1B*) forms the origin recognition complex 1, a component of DNA replication initiation complexes that is also implicated in transcriptional regulation^38^. The *orc1a* mutant was described by De la Paz Sánchez and Gutiérrez^38^ as having smaller cell size and an increased cell density in their cotyledon epidermis. This phenotype was also observed in *orc1b* plants while the double mutants *orc1a orc1b* were not viable. The *ORC1* rice homolog (*OsORC1*) seems to be involved in cell proliferation^39,40^, an essential part of growth. *PPD1* and its homologue *PEAPOD 2* (*PPD2*) both encode for plant-specific putative DNA-binding. The observed phenotype in *ppd1* plants contrasts with the one described by White^41^, who studying L*er*-0 mutants lacking *PPD1* function showed an excess in lamina growth while plants had a reduced lamina size when the gene was overexpressed. The difference could be explained by the natural accession in which the mutation was studied, being L*er*-0 in White^41^ and Col-0 in here. González et al.^42^ generated transgenic Col-0 plants carrying an artificial microRNA that targets both *PPD1* and *PPD2*. These plants with reduced transcripts of *PPD1* and *PPD2* also showed an increased leaf area^43^. We have observed that in wild-type Col-0 the disruption of only *PPD1* results in plants with a smaller rosette area, so the double mutant *ppd1 ppd2* may have an opposite phenotype than *ppd1* in Col-0. PPD1 has a TIFY domain, and in rice the overexpression of *TIFY* genes promotes plant growth^44^. Since we have observed that *pdd1* plants have a reduced growth, we speculate that *TIFY* genes also promote growth in *A. thaliana*.

Finally, we have identified two growth-related genes that have not been previously described: *MINICHROMOSOME MAINTENANCE 4* (*MCM4*) and *REMORIN 1.2* (*REM1.2*). *MCM4* encodes a minichromosome maintenance family protein, whose function is to ensure that DNA replication during S phase occurs completely and accurately^45^. It was described before that MCM proteins in *A. thaliana* and crop species (e.g., maize, pea and rice) are highly expressed in dividing tissues, as shoot apex and root tips^45^. For example, when *MCM2* is disrupted, plants are not viable due to problems during early embryonic stages meanwhile its overexpression increases cell division in root meristems^46^. Therefore, MCM4 has a role in the mitotic cycle and consequently in cell proliferation, which is key for plant growth^47^. Orthologs of this protein in other organisms also have significant roles in growth, as individuals of *Caenorhabditis elegans*^48^ or *Homo sapiens*^49^ with deficiencies in MCM4 suffer of slower growth. The *REM1.2* gene encodes a member of the remorin family of proteins whose function is not well understood but seem to have an important role in lipid rafts, platforms for signal transduction processes implicated in signaling^50^. It has been shown that remorins can restrict cell-to-cell movement of plant viruses^51,52^ or interact with symbiotic receptors to regulate bacterial infection in root nodules^53^. Hence, REM1.2 seems implicated in plant–microbe interactions. As an immunity-related receptor, its function may be also implicated in the plant growth signaling. This is because immunity and growth-related receptors share common co-receptors and signaling components^54^. REM1.2 may be a receptor also implicated in plant growth, either for its role in sensing growth-related signals or for its effects in binding growth-related signaling components that makes them less available. Despite growth being a trait controlled by many small-effect loci, we have been able to identify genes with a significative effect on the trait.

Overall, despite growth is controlled by many small-effect loci, we have been able to identify genes with a significant effect on the trait. In addition, some of these loci showed significant pleiotropic effects on other traits, increasing the complexity of the underlying genetic architecture^10^. Specially, it has been well documented the pleiotropy of genes affecting growth on flowering and *vice versa*^55–58^. Our GWAS was performed trying to minimize the effect of flowering initiation on *ELSA* determination. Yet, we found that, out of the four gene KO mutants with a significant alteration in growth, three had also an effect in flowering: *rem1.2*, *orc1a* and *ppd1* plants had a significant delay in their bolting time. As discussed before, REM1.2 may be a receptor for growth-related signaling components, which might include signals for the regulation of flowering initiation. This may also include signals important for initiation of flowering. The lack of this receptor may cause a delay in flowering. ORC1 has a PHD motif that binds H3K4me3, leading to an increase in H4 acetylation and H4K20 trimethylation^38^. Modifications in the chromatin regulate the expression of genes that control the time when the plant initiates flowering^59^. As mentioned above, PPD1 has a TIFY domain and it has been speculated that the TIFY domain may function in jasmonate (JA) signaling^60^, which has also been involved in flowering control^61,62^. Furthermore, other genes identified in this study have been previously associated with flowering phenotypes. In particular, although we did not find any effect of *abf3* on flowering time, previous studies have shown late flowering in *abf3* plants^63^ and early flowering in plants with ectopic *ABF3* expression in their vasculature^64^. The differences between these two studies and ours could be explained by the different phenotypes measured: both of the aforementioned studies evaluated flowering as the number of leaves at bolting while here we measured the day at which the transition happened. Therefore, some of the growth-related genes identified in this study also displayed pleiotropic effects on flowering time.

In summary, we have studied plant growth by performing a GWAS with the effective leaf surface area of a large number of natural accessions. The analysis of the data obtained from 710 natural accessions allowed us to identify 10 genes significantly associated with this trait. Various genes were further validated by studying how their disruption affected plant growth, finding that *mcm4*, *orc1a*, *ppd1*, and *rem1.2* mutants showed a reduction of their effective leaf surface area. Therefore, our GWAS has identified genes with a known development-related function (*ABF3*, *ORC1A*, and *PPD1*) and genes that were not previously linked to plant growth (*MCM4* and *REM1.2*). These results show that, despite growth is controlled by many genes, some of them showing pleiotropic effects on flowering, the GWAS could still identify genes with a significant effect on the rosette *ELSA*. This work corroborates the usefulness of smartphone-developed apps to perform GWAS and contributes to the knowledge of the genetics of the complex trait growth.

## Materials and methods

### Plant material and growth conditions

In this study we analyzed 710 wild accession of *A. thaliana* (Supplementary Table S1) differentiated by a total of 591,427 SNPs. Seeds of the 710 accessions were kindly provided by Prof. Magnus Nordborg (GMI, Vienna). In addition, the following SALK homozygous lines obtained from the Nottingham *Arabidopsis* Stock Centre (NASC) were analyzed: *AT1G75200* (SALK_076701C), *AT2G16440* (SALK_099676C), *AT3G61260* (SALK_024645C), *AT4G14700* (SALK_104400C), *AT4G14713* (SALK_057237C), and *AT4G34000* (SALK_138872C).

For GWAS, the 710 accessions were grown simultaneously in three experimental blocks, using a growth chamber set up with 16 h of light at 24 °C and 8 h of darkness at 20 °C, with a 45% RH and 125 µmol m^−2^ s^−1^ of light intensity. Before seed sowing, seeds were stratified at 4 °C for 2 days to synchronize germination. Two plants per accession were grown and measured in the same pot. To reduce variation caused by the position of the plant in the growth chamber, pots were moved to new position every three days. For validation of genes, the wild-type Col-0 and mutant plants were grown similarly, but including between eight and ten plants per genotype, with the plants being grown in individual pots. The position of the pots was randomly changed every.

### Determination of plant phenotypes

To measure the effective leaf surface area (*ELSA*) we used Easy Leaf Area smartphone app^23^ to process plant rosette images. If the plant had flowered, the stem was removed in order to capture only the area of the rosette. To evaluate the KO mutants, measurements were performed in intervals of 3 days. Bolting time was evaluated daily by visual inspection of plants and determined as the number of days from sowing to the start of stem elongation. Stem plant height was measured with a ruler and the day of bolting by visual inspection. Statistical analyses of the phenotype measurements were done using SPSS version 25 (IBM Corporation, Armonk NY, USA).

### GWAS analysis

The analysis was performed using a Genome-wide Efficient Mixed Model Association algorithm^25^ (GEMMA). The genotype data was obtained from https://1001genomes.org/data/GMI-MPI/releases/v3.1/ in a vcf format. Using PLINK 1.9^65^. we recoded the vcf format into the PED binary format and removed SNPs on the basis of missing genotype rate, including only those with ≥ 95% genotyping rate. The genotype files were also filtered to keep only the information for the 710 accessions we phenotyped. We used the centered relatedness matrix to account for population structure. The *ELSA* trait was standardized by flowering using a univariate general linear model in SPSS and run in GEMMA with a minor allele frequency cut-off set to 5%. We used likelihood ratio *P* values from the GEMMA output files because likelihood ratio test makes fewer approximations compared to the Wald’s test. Manhattan and QQ-plots were drawn using rMVP package^66^ in R version 3.6.1 in RStudio version 1.2.1335.

### Bayesian sparse linear mixed model (BSLMM)

To study the genetic architecture of the *ELSA* we used BSLMM implemented in GEMMA^25,67^. We analyzed the leaf area phenotype using the linear BSLMM Markov chain Monte Carlo (MCMC) with default settings (burn-in at 100,000, sampling steps at 1,000,000 and recording every ten steps) and minor allele frequency cut-off set at 5%.

## Supplementary information

Supplementary Table S1

Supplementary Table S2

Supplementary Table S3

## Data Availability

The data generated or analyzed during this study are included in this published article as Supplementary Information files.
